# Yield and Yield Components in Soybean Using Genome-Wide Association Studies, GWAS: Current Status, Challenges, and Breeding Prospects

**DOI:** 10.3390/plants15203243

**Published:** 2026-10-21

**Authors:** Aigul Amangeldiyeva, Svetlana Didorenko, Bekzhan Maikotov, Assel Jenisbayeva, Yuri Shavrukov, Raushan Yerzhebayeva

**Affiliations:** 1 Plant Biotechnology Laboratory, Kazakh Research Institute of Agriculture and Plant Growing, Almalybak 040909, Kazakhstan; aigul_seidinabiyeva@inbox.ru (A.A.); svetl_did@mail.ru (S.D.); maikotov.bekzhan@mail.ru (B.M.); jenisbayeva@gmail.com (A.J.); 2 Department of Biology and Biotechnology, Farabi University, Almaty 050040, Kazakhstan; 3 College of Science and Engineering, Biological Sciences, Flinders University, Adelaide, SA 5042, Australia; yuri.shavrukov@flinders.edu.au

**Keywords:** soybean, water deficit, drought tolerance, yield, yield components, genome-wide association study (GWAS), candidate gene, marker-assisted selection (MAS), biological pathway

## Abstract

Molecular-genetic analysis of soybean yield and yield components under drought is a crucial element for the development of effective breeding strategies and sustainable agricultural production. The aim of this review is to critically analyse current data from genome-wide association studies (GWAS) and identified genetic loci, SNP markers, and candidate genes associated with yield components in soybean in dry conditions with limited water supply and adequate watering (controls). In the results, 346 genetic associations, and 229 candidate genes with KEGG-based potential functions were sufficiently described in published papers and selected for the current study. Three statistically significant metabolic pathways were identified with isoflavonoid biosynthesis, glycosaminoglycan degradation and cysteine and methionine metabolism. The main limitation of the presented analysis was found in the low level of heritability for the studied traits, together with human factors such as carrying out and interpreting GWAS affecting the results presented in the current study. Additionally, complex multigenic controls were reported for the studied traits in soybean and a high level of genotype × environment interactions. Nevertheless, the presented data provide a basis for developing molecular strategies to improve soybean yield in conjunction with better potential drought tolerance.

## 1. Introduction

Human population growth and climate change pose serious challenges to global food security [1,2]. Rising temperatures, uneven distribution of precipitation, and more frequent droughts are reducing crop productivity. On current trends, a 1 °C increase in mean global temperature may reduce the yield of major crops by 3.1–7.4%, while drought already accounts for up to 30% of annual global crop losses [3,4]. Accordingly, developing high-yielding cultivars with improved tolerance to water deficit is one of the priority directions in modern crop breeding.

Soybean [*Glycine max* (L.) Merr.] is one of the most economically important crops worldwide and plays a key role in ensuring food and feed security. Soybean seeds contain approximately 40% protein and 20% oil, which accounts for its widespread use in the food, feed, and processing industries [5,6,7,8,9,10]. In addition, soybean serves as an important feedstock for biodiesel production [11,12] and, as a nitrogen-fixing legume, contributes to maintaining soil fertility and reducing the need for mineral nitrogen fertilizers [12]. The expected growth in global demand for soybean, driven by population growth and increasing consumption of plant-based protein, further underscores the importance of this crop [13].

Drought is one of the major constraints on soybean production, as the crop exhibits high sensitivity to water deficit [14,15,16,17,18]. Optimal soybean growth and development require approximately 450–700 mm of precipitation during the growing season. However, climate change is intensifying water deficit in soybean-growing regions [2,19,20]. Drought accounts for about 12.8% of the annual variation in soybean yield. Under water deficit, yield losses can reach 40–80%, depending on genotype and developmental stage [21,22,23,24]. The germination, flowering, and seed-filling stages are the most critical, as water stress during these periods reduces germination rate, seed number, and ultimately yield [4,25,26,27,28,29]. Thus, improving soybean drought tolerance requires a deeper understanding of the genetic control of yield and its components under differing water supply.

The effects of drought can ultimately reduce seed yield, which is the main cause of economic losses in soybean production. Yield is therefore the most challenging trait for breeders to improve. However, direct breeding for yield is difficult for three main reasons: its complex genetic control (many genes with small effects), strong genotype × environment interactions, and epistatic effects [30]. For this reason, particular attention is given to yield components, which can serve as indirect selection criteria. Researchers typically analyse yield components such as the number of seeds per plant and seed weight per plant, rather than evaluating yield directly. This strategy has proven effective, as these components have a simpler genetic control and are less influenced by environmental factors [31]. Such approaches have formed the basis of many studies on drought tolerance in soybean for yield components used as criteria [32,33,34,35].

Drought tolerance is a complex quantitative trait controlled by numerous genes interacting additively, dominantly, and epistatically [36,37,38,39,40]. The mapping of quantitative trait loci (QTL) is one of the main approaches used to study the genetic control of drought tolerance [41]. The use of biparental populations has enabled the identification of multiple QTLs associated with yield components, including pod number, biomass, and seed weight per plant [42,43,44]. These studies have substantially contributed to our understanding of the genetic basis of productivity in crops such as maize [45,46], rice [47], soybean [48,49,50], and others.

According to the SoyBase database [51], hundreds of QTLs associated with yield and its components have been mapped across the soybean genome to date. However, traditional linkage mapping has several limitations. These include low allelic diversity restricted to the two parental lines and the lengthy process of developing mapping populations. Mapping resolution is also relatively low, which hinders precise localization of causal genes [49,52,53].

The limitations of traditional linkage mapping have driven the widespread use of genome-wide association studies (GWAS), which exploit the natural genetic diversity present within populations [54,55,56]. Like QTL mapping, GWAS aims to identify loci and alleles controlling phenotypic variation, but it offers several notable advantages. The use of high-density single-nucleotide polymorphism (SNP) markers provides higher mapping resolution and allows for more precise localisation of candidate genes [57]. Advances in next-generation sequencing technologies have made GWAS one of the most powerful tools for studying the genetic structure of complex quantitative traits and a cornerstone of modern genomics-assisted breeding [57,58,59,60]. The effectiveness of this approach has been demonstrated in identifying QTLs and candidate genes associated with protein and oil content, agronomic traits, salt tolerance, and yield components in soybean [50,58,59,60].

Accordingly, the analysis of GWAS findings is becoming an important tool for understanding the genetic control of soybean yield components under drought conditions. However, a review of the published literature reveals a substantial imbalance. Most GWAS studies of soybean yield components have been conducted under optimal water supply (irrigated or well-watered conditions) [61,62,63,64,65], which eliminates the influence of soil drought and allows the genetic potential for productivity to be assessed under favorable conditions [30,66]. As a result, databases such as SoyBase [51] have accumulated hundreds of QTLs for seed weight, pod number, and other traits, yet the effectiveness of these loci under actual water deficit conditions remains largely unexplored. Only a limited number of studies have addressed GWAS analysis of soybean yield components under natural or artificially induced drought [67,68,69]. Moreover, the published studies differ considerably in the plant material used, research methodology, and experimental conditions. Thus, it remains unclear to what extent the genetic control of soybean yield established under ‘irrigated’ conditions also holds under drought, and whether the same genetic associations (SNPs/QTLs) are involved or drought instead activates fundamentally different genetic mechanisms that are not expressed under well-watered conditions. Answering this question is critical for developing effective markers for drought-tolerance breeding, since simply extrapolating markers selected for irrigated fields to rainfed conditions may prove ineffective and economically unjustified.

Therefore, a comprehensive analysis of published GWAS studies is needed in order to systematize the identified genetic associations with yield and its components in soybean; to compare the results obtained under drought and well-watered conditions, assess their reproducibility across studies and environments, examine the evidence supporting candidate genes and the criteria used to select them in the original studies, and determine the extent to which these findings have been translated into validated molecular markers for practical use in breeding.

This review aims to critically analyse the available GWAS results for soybean yield components obtained under different water regimes, including optimal irrigation and water-deficit conditions. It also assesses the reproducibility of the identified genetic associations, and candidate genes. In addition, the review examines the main factors underlying discrepancies among studies and outlines some promising directions for the application of GWAS in soybean breeding.

## 2. Methods for Analyzing Published Data

This article is a narrative review that combines a comprehensive, interpretative analysis of the scientific literature with a bibliometric analysis of publications. It summarizes and synthesizes existing GWAS of yield and yield components in soybean under drought and well-watered conditions. In addition, the genes and loci reported in the published GWAS were compiled into datasets and subjected to enrichment analysis to identify the biological processes and functional categories represented among the identified candidates.

The methodology of this study comprised a bibliometric analysis of the relevant publications and a functional enrichment analysis of candidate genes. The bibliometric search was performed in the Scopus database [70] in August 2026. A search of the Scopus database using the keywords “soybean” and “genome-wide association study” retrieved 1069 publications, reflecting the overall body of research on the application of GWAS in soybean. Adding the keyword “drought” narrowed the results to 120 publications. This narrower search was used specifically for the bibliometric analysis of drought-related GWAS research in soybean and did not define the inclusion criteria for the subsequent compilation of genetic associations. The resulting publications were exported in CSV format and analyzed using VOSviewer, version 1.6.21 [71]. Note that, although Scopus was the primary database for the bibliometric analysis, the literature search for this review was not limited to Scopus. Additional publications and relevant information were identified through other scientific databases and search platforms, including Web of Science, Google Scholar and PubMed. These sources supplemented the Scopus search and helped identify relevant studies that were not captured in the bibliometric dataset. The bibliometric analysis characterized the overall volume, structure and main research directions of GWAS in soybean, with particular attention to yield, yield components and drought tolerance. Its results provided an overview of research trends and the context for the subsequent analysis of relevant publications.

Importantly, the bibliometric search strategy and the literature selection process for the compilation of GWAS associations were treated as separate procedures. While the bibliometric analysis focused on drought-related GWAS publications, the literature review considered studies reporting genetic associations with soybean yield and yield components under different environmental conditions, including studies conducted exclusively under well-watered (WW) conditions. Therefore, the inclusion of publications in the GWAS data compilation was not restricted to studies containing the keyword “drought”.

Following the literature search, publications were selected based on their relevance to the scope of this review. The selection process included soybean GWAS studies reporting genetic associations with yield and yield components under well-watered (WW), drought stress (DS), or both conditions. Importantly, in studies investigating drought stress, well-watered (WW) conditions were generally included as a control treatment, allowing the comparison of genetic associations and yield-related traits between WW and DS conditions. Studies conducted exclusively under WW conditions were also eligible for inclusion, even when no corresponding DS data were available. For each publication, we extracted information on the reported genetic associations, the chromosomes and physical positions of the loci, the traits studied, the environmental conditions, the GWAS models used and any candidate genes proposed by the authors.

Following the examination of the retrieved literature, 16 GWAS studies were selected for detailed data compilation based on their direct relevance to soybean yield and its components. These studies provided the candidate-gene and genetic association data used for the subsequent analyses. The final datasets comprised 229 candidate-gene records representing 216 unique gene identifiers (Supplementary Table S1) and 346 genetic association records corresponding to 339 unique association IDs (Supplementary Table S2). Some candidate genes were associated with different traits or genetic markers, while certain association IDs were reported for multiple traits. To visualise the overall distribution of genomic regions associated with yield and yield components in soybean, we used the physical positions of SNPs, QTNs, and QTLs as originally reported in the respective published GWAS studies. Since these studies were conducted using different versions of the soybean reference genome, the original genomic coordinates were retained without standardisation to a common reference assembly. The resulting chromosomal map was intended to provide a descriptive overview of published genetic associations rather than a precise assessment of physical co-localisation across studies. SNPs, QTNs, and QTLs were distinguished according to their original classification and were not combined into a single statistical category. For functional characterisation, candidate gene identifiers were standardised against the Glycine max Williams 82 reference genome (Wm82.a4.v1). Kyoto Encyclopedia of Genes and Genomes (KEGG) [72] pathway enrichment analysis was performed using ShinyGO 0.86.0 [73]. All protein-coding genes of the soybean genome available in the ShinyGO annotation database were used as the background gene set, as no custom background was specified. For each pathway, the number of candidate genes, fold enrichment, and false discovery rate (FDR) were determined. Enrichment was considered statistically significant at FDR < 0.05.

Generative AI tools were used to improve the clarity and language of the manuscript and to assist with the organisation and structure of Section 7. All suggestions were critically reviewed, verified, and revised by the authors before inclusion. The tools were not used for literature analysis, data generation, or the formulation of scientific conclusions.

## 3. Soybean Yield as the Primary Breeding Trait and Its Components

### 3.1. Soybean Yield as the Primary Indicator of Breeding Efficiency and Evaluation of Drought Tolerance

In soybean plants, individual components of yield directly determine the final productivity and may respond differently to water deficit [74,75]. Soybean international germplasm collections are also evaluated primarily on the basis of these traits, which makes it possible to more effectively identify promising donors of agronomically valuable alleles and to accelerate the breeding process [76].

Soybean yield formation begins during the vegetative phase, when the main structural elements of the plant (leaves, stems, and nodes) are established, providing the basis for subsequent biomass accumulation [74]. During the reproductive period, the main yield components are formed, namely pod number, seed number, and seed weight, which together determine final plant productivity [77,78]. Different components are formed at subsequent developmental stages and may, therefore, respond differently to changes in environmental conditions, including water deficit [79].

### 3.2. Main Soybean Yield Components and Their Interrelationships

The main components of soybean yield (Y) include seed weight per plant (SWP), number of productive nodes (NPN), pod number per plant (PNP), number of seeds per pod (NSP), and 100 seed weight (HSW), which is sometimes referred to as seed weight (SW), [74,80,81]. HSW or SW more often plays a compensatory role and can vary depending on growing conditions [69,74]. Multiple studies show that the increase in yields achieved in modern soybean cultivars is largely attributable to higher NPN and PNP. For example, authors [82] found that the yield improvement of cultivars developed over recent decades was accompanied by an increase in the number of nodes and pods. Similar results of a consistent increase in NPN and PNP were reported in cultivars released between 1920 and 2010 [83]. These traits reflect the efficiency of the physiological processes underlying yield formation regarding key components of plant productivity.

Many studies confirm close positive correlations between yield components and final yield. Strong positive relationships have been established between seed yield and harvest index, HSW, NSP, and biological yield [84]. According to path coefficient analysis, PNP shows the greatest direct positive effect on seed yield [85]. Biological yield (r = 0.96), harvest index (r = 0.92), and branch number (r = 0.92) also show high positive correlations with yield. At the same time, the relationships between components may be compensatory in nature. For example, under stress conditions, an increase in HSW is sometimes accompanied by a decrease in seed number, and vice versa. Studying correlations and path coefficient results make it possible to identify target traits that have the most substantial direct and indirect effects on final yield, and to use them as effective selection criteria [29].

Drought negatively affects all of the above-mentioned components. Water deficit during flowering and pod formation reduces pod set and leads to abortion of reproductive organs, decreasing seed number by 13–64% [80]. During the seed-filling stage, drought shortens the duration of this phase and reduces seed weight, although seed number may remain unchanged [81]. Notably, the effect of drought on yield-structure elements is not uniform and depends on both genotype and the intensity and duration of the stress [83]. Under water deficit, some genotypes may show increased NSP that is accompanied by decreased SW, while in some cases, SW can be unchanged but NSP is reduced [29,83]. This variability in response reflects genetic differences in mechanisms of adaptation to drought and creates breeding opportunities for better drought tolerance [86].

Water deficit during the seed-filling stage directly limits plant photosynthetic productivity. It also disrupts the transport and redistribution of assimilates within the ‘leaf-pod-seed’ system and, consequently, both SNP and SW are reduced. Insufficient supply of photoassimilates to developing seeds reduces their capacity to accumulate carbohydrates, proteins, and lipids, resulting in a lower seed-filling rate and reduced seed size and final seed weight. Thus, the drought-induced decline in soybean yield results from the combined effects of stress on photosynthesis, assimilate formation and transport, ‘source-sink’ relationships, and the processes of seed formation and filling [87,88].

### 3.3. Genetic Control of Yield Components and Their Practical Significance for Breeding

Yield and yield components have a polygenic basis. However, individual components may show higher heritability and lower phenotypic variability. In particular, HSW, PNP and NPN have shown higher heritability than yield in some studies [30,89]. The genetic effects can also depend on environmental conditions [67,68]. Studying yield components therefore allows a more detailed assessment of the genetic factors underlying soybean productivity and facilitates the identification of stable genetic associations with potential for breeding applications [67].

The efficiency of determined genetic factors also depends on the diversity of the genetic resource material. Wild soybean (*Glycine soja*), landraces, and modern cultivars differ in the level and structure of their genetic diversity and, therefore, represent valuable sources of genetic polymorphism for agronomically important traits. *G. soja* and landraces may have rare and adaptive alleles, whereas modern cultivars have primarily been selected for high productivity and other agronomically valuable traits under favourable conditions. The use of different germplasm generates broader genetic variability and increases the probability of identifying novel loci and alleles associated with yield and drought tolerance [89].

## 4. Principles of GWAS and Their Application in the Genetic Control of Soybean Yield Components Under Drought Conditions

**Figure 1:**
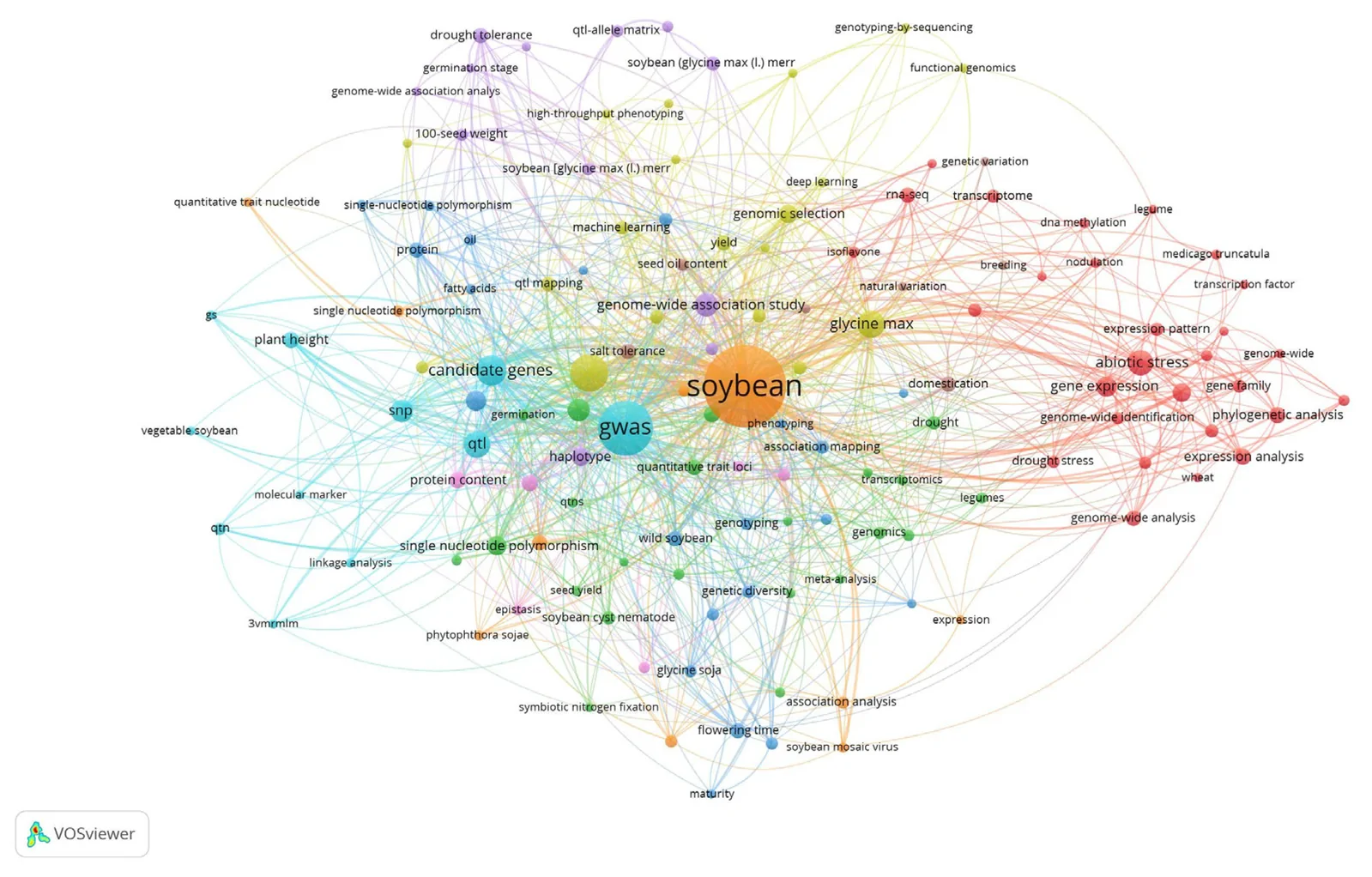
Keyword co-occurrence network of soybean GWAS research generated using VOSviewer. Node size indicates keyword occurrence frequency, lines represent co-occurrence relationships, and colors distinguish thematic clusters.

The keyword co-occurrence analysis performed using VOSviewer provided an overview of the main research directions in soybean GWAS studies (Figure 1). The bibliometric network comprised 136 terms grouped into nine thematic clusters. The most frequently occurring terms included “soybean” (412 occurrences; total link strength [TLS] = 872), “GWAS” (182; TLS = 428), “genome-wide association study” (85; TLS = 182), “candidate genes” (57; TLS = 155), “QTL” (43; TLS = 121), and “SNP” (31; TLS = 75).

The thematic structure of the network reflected several interconnected research areas, including gene expression and functional genomics, abiotic stress responses, SNP and QTL mapping, genetic diversity and association mapping, genomic selection and advanced computational approaches, haplotype analysis, and molecular and marker-assisted breeding. At the same time, the clustering patterns suggested a degree of thematic separation between the identification of genetic associations and their functional interpretation. Terms related to gene expression, RNA-seq, transcriptomics, and abiotic stress formed a distinct thematic group, whereas major terms associated with GWAS, QTLs, and SNPs were concentrated in other clusters (Figure 1). This pattern highlights the importance of closer integration of association mapping with functional genomic approaches to validate the biological roles of identified candidate genes.

Thus, the VOSviewer analysis provided a broader bibliometric context for this review and highlighted the relevance of integrating GWAS findings with functional genomic evidence for the interpretation and validation of candidate genes associated with soybean yield, yield components, and drought tolerance.

At present, GWAS is one of the most effective methods for identifying genetic loci controlling complex quantitative traits in crops. The method is based on detecting statistically significant associations between SNPs and phenotypic variation [90,91].

The effectiveness of GWAS depends on the size of the study population, its level of genetic diversity, the accuracy of phenotyping, and the density of molecular markers. The analysis comprises several consecutive steps: assembling a representative panel of genotypes (1), phenotypic evaluation of traits (2), high-throughput genotyping (3), SNP marker quality control (4), assessment of population structure and the degree of relatedness among accessions (5), statistical association analysis (6), and the subsequent identification of significant SNPs (7), quantitative trait nucleotides (QTNs) (8), Quantitative trait loci (QTLs) (9), and candidate genes (10) [91,92] followed by their validation (11) and, where marker effectiveness is confirmed, application in marker-assisted selection (MAS) (12) [65].

**Figure 2:**
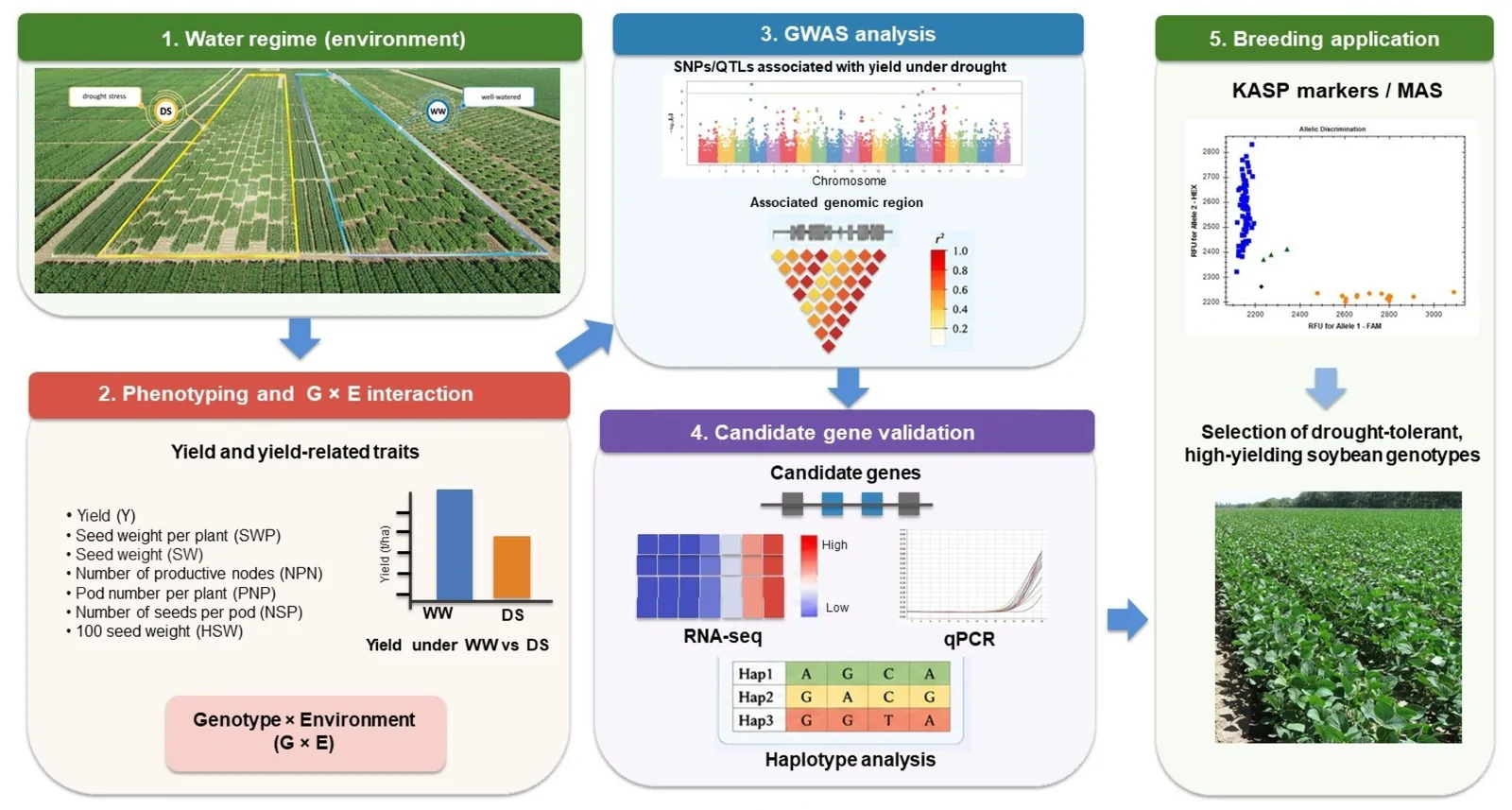
Conceptual framework linking water regime, G × E interaction, GWAS, candidate-gene validation and breeding application. (1) Reproduced from [67]; (2–5) created by the authors.

Medium- and high-density SNP chips (SoySNP50K Illumina iSelect BeadChip, Illumina SoyNAM BeadChip, NJAU 355K SoySNP Array) are widely used for genotyping in soybean studies, along with modern high-throughput sequencing technologies, including GBS (genotyping-by-sequencing), SLAF-seq, DArTseq, Illumina HiSeq, Illumina HiSeq 2500, BGISEQ-T7, and whole-genome sequencing (WGS). These technologies allow the detection of tens of thousands up to millions of SNP markers, substantially increasing the resolution of GWAS and the accuracy of candidate gene localization [67,68,69] (Figure 2).

One of the key requirements for reliable GWAS results is controlling for false-positive associations arising from population structure and genetic relatedness among accessions. Mixed linear models (MLM), which account for population structure (PCA or the Q-matrix) and the kinship matrix, are the most widely used approach for addressing this issue [93]. In recent years, the multi-locus models FarmCPU (Fixed and Random Model Circulating Probability Unification) and BLINK (Bayesian-information and Linkage-disequilibrium Iteratively Nested Keyway) have become increasingly popular, as they offer higher statistical power, reduce the number of false positives, and provide more accurate identification of loci controlling complex quantitative traits [71,94]. Alongside these, haplotype-based GWAS, gene-centric GWAS, and fine-mapping methods are being actively developed, allowing more precise localization of causal variants and improving the efficiency of candidate gene discovery [91].

Despite the rapid development of GWAS, a review of the published literature shows that most studies identifying loci associated with soybean yield have been conducted under well-watered conditions. Large-scale studies of HSW, PNP, biomass, and total yield have predominantly been carried out under optimal hydrothermal conditions, which reduces the influence of environmental factors and increases the heritability of traits studied [58,59,60]. In contrast, field studies specifically designed to simulate drought using natural or controlled water deficit remain relatively scarce [67,68,69]. This substantially limits the practical use of the identified SNP markers in breeding, since loci detected under optimal water supply do not always retain their significance under stress conditions.

Accordingly, studies conducted across multiple environments and water-supply regimes, using genetically different collections that include modern cultivars, landraces, and wild *G. soja* accessions, are of particular value. Such an approach makes it possible to identify stable, stress-specific QTLs and candidate genes with minimal genotype × environment interaction. It can considerably increase their potential for use in marker-assisted selection (MAS) for developing high-yielding and drought tolerant soybean cultivars [66,67,68].

Before examining the effects of drought on yield and its components, it is useful to analyse the scientific literature on soybean GWAS in order to assess the geographical distribution of these studies, the populations used, and their main research directions.

## 5. Main GWAS Findings and Factors Determining Their Effectiveness

In recent decades, GWAS have been widely used to investigate the genetic basis of complex, agronomically important traits in various crops [95,96,97]. This approach has enabled the identification of many genomic regions, genetic associations, and candidate genes associated with agronomically important traits, substantially expanding our understanding of the genetic control of quantitative traits. However, detecting a statistically significant association between a genetic variant and a phenotypic trait is not sufficient evidence of a causal role for a specific gene [98,99]. Candidate genes identified through GWAS therefore require subsequent independent functional validation to confirm their direct involvement in the trait of interest and to assess the potential for practical use of the identified associations in breeding [100].

A substantial body of scientific literature has now accumulated on the application of GWAS to study the genetic basis of various traits in a wide range of plant species, including *Arabidopsis* [90], rice [101], wheat [102], maize [103], soybean [104], and other crops. The use of GWAS is not limited to cereal crops and extends to a broad range of species, including cotton [105,106,107], tomato [108,109,110], sesame [111], peanut [112], lettuce [113], peach [114], and many others. This section presents key publications from 2014 to 2025 on the application of GWAS to study the genetic basis of soybean yield and its components. One of the earliest GWAS studies in soybean identified 19 SNPs and 5 haplotypes associated with yield and its components across several environments. Among these, haplotype hp40, comprising two linked SNPs, was localised within the region of a putative causal gene, *Glyma11g29460* [115], which encodes cinnamoyl-CoA reductase (CCR). This enzyme is involved in lignin biosynthesis, which plays an important role in cell wall formation and in the functioning of the xylem, which is responsible for water and nutrient transport [116]. *Glyma11g29460* was, therefore, proposed as a potential candidate gene involved in high soybean yield and its components.

Data on the genetic loci and candidate genes associated with yield traits and their components were systematised based on the analysis of published GWAS studies. For each selected study, information on the identified genetic associations was extracted. Candidate genes were included only when they had been explicitly identified or proposed by the authors of the respective publications. Candidate gene identifiers, their association with the phenotypic traits studied, and information on the corresponding genetic associations and chromosomal locations were extracted from the relevant publications (Supplementary Table S1). Consequently, candidate genes were not available for all identified associations. This synthesis of published data yielded a set of 229 candidate genes identified across various soybean GWAS studies. This gene set was subsequently used for functional characterization and to identify potentially related biological processes and metabolic pathways.

**Figure 3:**
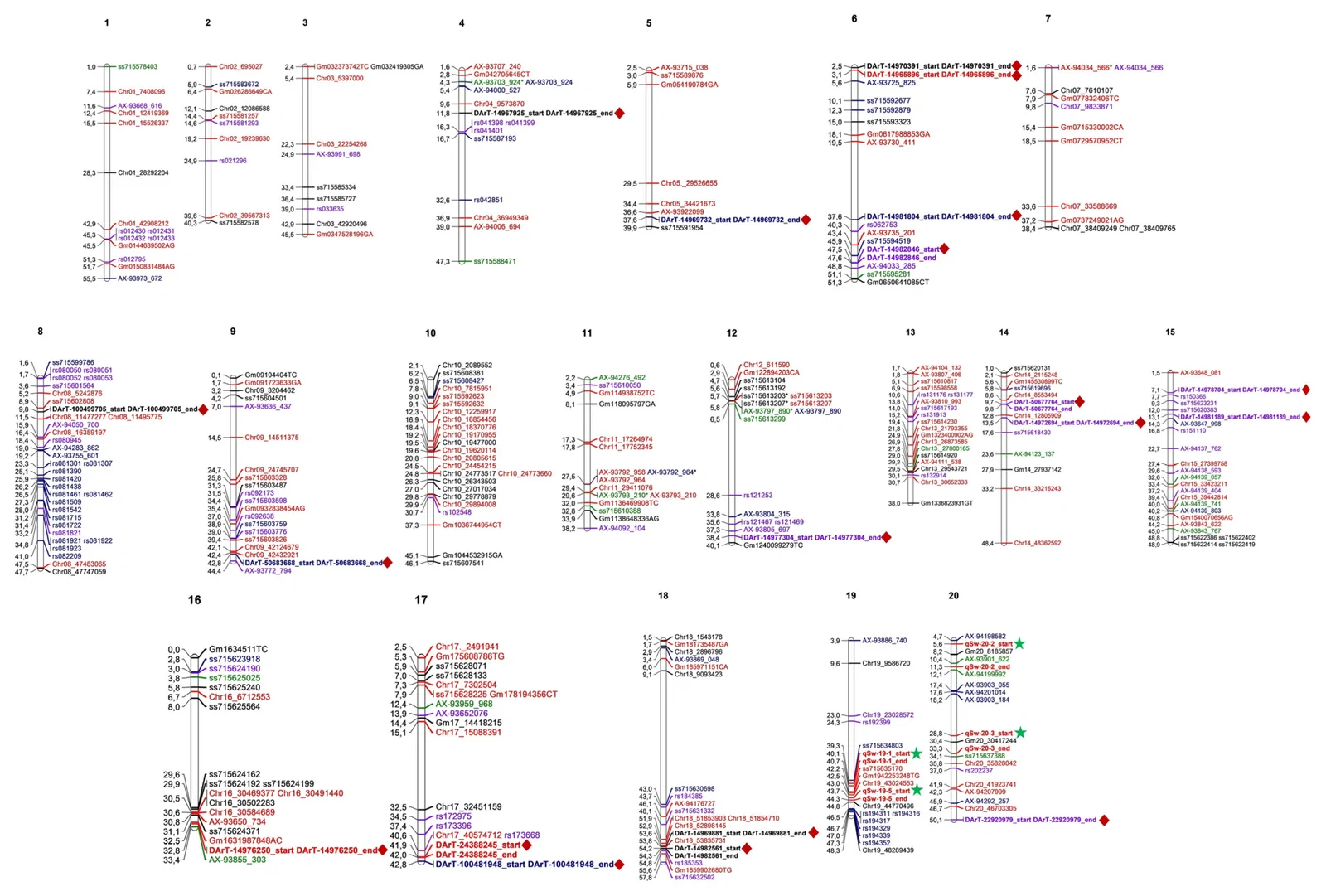
Distribution of molecular markers and genetic loci based on the current analysis across 20 chromosomes in soybean (*Glycine max* L.). Colour of the encoded traits are as follows: black, yield (Y); purple, seed weight per plant (SWP); red, 100 seed weight (HSW); blue, pod number per plant (PNP); and green, number of seeds per pod (NSP). QTLs are indicated by green asterisks, whereas QTNs are indicated by red rhombus. The start and end positions are provided for each QTL and QTN. * indicates a QTL or SNP associated with two traits. Details of the presented markers and loci are provided in Supplementary Table S2. The map was constructed using program MapChart, version 3.32 [117].

Despite the high effectiveness of GWAS in identifying genetic loci controlling yield and its components, results differ substantially among studies. The size and genetic diversity of the study population, growing conditions, genotyping density, phenotypic data quality, and statistical models used all influence the number of detected QTLs, SNP markers, QTN (genetic associations) and candidate genes, as demonstrated by the analysis of published data [30,61,62,63,64,65,67,68,69]. Consequently, the practical value of the identified associations is determined not only by their statistical significance but also by their reproducibility across independent populations and different environmental conditions. However, our analysis of the reviewed publications showed that none of the identified genetic associations had been detected again in independent studies or populations. This indicates limited evidence of their reproducibility and highlights the need for further independent validation. (Figure 3 and Supplementary Table S2).

Based on the analysis and systematisation of data from published GWAS studies, a total of 346 genetic associations related to soybean yield and yield components were compiled, including 323 SNPs, 19 QTNs, and 4 QTLs (Figure 3 and Supplementary Table S2). Their distribution across chromosomes was visualised using the online program MapChart, version v3.32 [117] with original data presented in Supplementary Table S2. Marker distribution across chromosomes was uneven, with their number and density varying significantly between individual chromosomes. The highest number of markers was localised on chromosome 8 (34 SNPs and QTLs), while only 8 associations were found on chromosome 5. On chromosome 8, 18 genetic associations related to PNP were reported within the approximately 18–42 Mb region according to their original physical positions. However, because the reported coordinates were derived from studies using potentially different reference genome assemblies, this apparent concentration should be interpreted cautiously and does not necessarily indicate precise physical co-localisation. The reported associations and candidate genes suggest that chromosome 8 may contain genomic regions of interest for further investigation of the genetic control of PNP. Nevertheless, coordinate standardisation and functional validation are required to confirm the physical relationships between these loci and their potential biological roles. Nevertheless, coordinate standardisation and functional validation are required to confirm the physical relationships between these loci and their potential biological roles. Twenty-four genetic associations were localised on chromosome 10, and nine of these markers were associated with Y. Two candidate genes for Y identified on chromosome 10 included *Glyma.10G219500* and *Glyma.10g023900*. Markers associated with SWP were primarily localised on chromosomes 8, 9, and 15, with 8, 6, and 9 markers, respectively. Genetic associations with HSW were mostly represented on chromosomes 10 and 13. The smallest number of associated markers was identified for NSP.

Additionally, genetic associations simultaneously associated with two traits of yield components were identified in several chromosomes. On chromosome 8, common markers were associated with Y and SWP (DArT-100499705), and on chromosome 10, with Y and HSW (Gm1044532915GA). On chromosomes 11, 14, and 20, markers simultaneously associated with NSP and PNP (AX-94276,492, AX-94123,137 and AX-94199992) were identified. On chromosome 13, a common association was observed for HSW and NSP (AX-94104,132 and AX-93807,406), while on chromosome 18, it was for HSW and PNP (AX-94176727). The presence of genetic associations associated with two different traits indicates for the possible existence of common genetic factors involved in yield component determination. Such associations may be due to the pleiotropic effect of individual loci or the close linkage of the identified genes.

Overall, the combined analysis of published genetic associations and candidate genes revealed an uneven distribution of reported associations across the soybean chromosomes. Certain chromosomes, particularly chromosome 8, contained a relatively high number of associations related to specific yield components, including PNP. However, because physical positions were retained as originally reported and were not standardised across reference genome assemblies, the apparent clustering of associations should be considered descriptive rather than evidence of precise genomic colocalisation.

Among the key factors determining the effectiveness of GWAS are the size of the study population and its level of genetic diversity. Increasing the number of genotypes analyzed increases the statistical power of the study and the likelihood of detecting loci with small effects. The density of molecular markers plays an equally important role. The use of modern genotyping technologies such as GBS, SLAF-seq, DArTseq, and high-density SNP arrays considerably increases mapping resolution compared with earlier studies based on SNP chips with a limited number of markers [61,63,67,68].

The choice of statistical model also has a substantial effect on the results. MLM, FarmCPU, and BLINK are the most widely used models in current studies, as they more effectively account for population structure and the degree of relatedness among genotypes, thereby reducing the number of false-positive associations. For this reason, many studies apply several GWAS models at the same time, and loci consistently detected by multiple statistical approaches are considered more robust to model choice within the analyzed dataset [61,62,63,67]. Notably, none of the identified loci showed independent replication across the reviewed studies or environments. The loci shown in Figure 2 should therefore be regarded as reported associations rather than independently replicated loci.

One of the limitations of GWAS is obtaining high-quality phenotypic data. Automated phenotyping using computer vision and deep learning improves the accuracy and reproducibility of trait evaluation, thereby contributing to more effective detection of genetic associations [118,119,120].

## 6. GWAS of Soybean Yield Traits Under Optimal Growing Conditions and Water Deficit

### 6.1. Yield

Although the genetic basis of yield formation has been extensively studied in cereal crops, the corresponding mechanisms underlying soybean productivity remain considerably less well understood [121]. One of the earliest approaches to studying the genetic factors controlling soybean yield was association analysis using SSR markers. A study of a wild soybean (*Glycine soja*) population showed that natural genetic diversity is a valuable source of alleles associated with plant productivity [122]. Although stable markers (sct_010 and satt316) associated with Y were identified, the limited density of SSR markers did not allow for precise localization of the causal genes. This limitation led to the use of high-density SNP platforms and GWAS methods. GWAS has repeatedly proven effective in identifying molecular markers associated with soybean Y. In a study of 219 soybean accessions, four SNPs were found to be statistically significant in association with Y across different growing conditions. The candidate genes *Glyma.13g073900*, *Glyma.06g050300*, *Glyma.03g169700*, and *Glyma.03g171900* were identified near these loci [123]. In another study of 169 soybean accessions genotyped with the BARCSoySNP6K SNP chip, six significant SNPs associated with Y were detected, four of which were located on chromosome 12 [124]. Six loci associated with this trait were identified in yet another study [122], whereas another study [125] revealed two statistically significant SNPs associated with Y, located on chromosomes 11 and 16.

The GWAS results accumulated to date indicate that the loci controlling soybean Y are distributed across almost the entire genome. According to the SoyBase database [51], more than 365 QTLs associated with seed yield have been identified. For example, authors [58] have detected SNP markers on chromosomes 14, 17, and 20, whereas another study [126] identified 23 Y-associated loci located on 16 chromosomes. Despite the large number of associations detected, only a few were accompanied by the identification of candidate genes with a plausible biological function. In particular, *Glyma.09g001300* is considered a promising candidate gene, as it is located near an SNP associated with both Y and maturity time. This gene shows high homology to an *Arabidopsis thaliana* transcription factor involved in regulating the far-red light response [127,128,129], suggesting a possible role in regulating maturity time and, indirectly, in Y formation. In addition, the detection of an SNP on chromosome 19 near a locus previously [130] indicates that some associations are reproducible across independent populations and different growing conditions. A three-year study identified 23 SNPs associated with soybean Y. Of these, only the loci on chromosomes 7, 8, and 10 were simultaneously associated with both HSW and Y. Twelve statistically significant SNPs associated with both traits were identified on chromosome 10 [63].

Over time, the research focus has gradually shifted from identifying as many associations as possible to assessing their reproducibility and interpreting their functional significance. For example, only two loci on chromosomes 9 and 17 with 19 SNPs associated with Y, retained a statistically significant effect across most of the environments examined [62]. The candidate genes *Glyma.09g048900*, *Glyma.17g090200*, and *Glyma.17g090400* were proposed for these regions. These findings demonstrate that stable loci retaining their effect across different growing conditions are of the greatest practical value for breeding, whereas most of the detected associations are environment-specific. At the same time, functional annotation of these loci can help explain the possible molecular mechanisms underlying yield formation. For instance, authors identified seven SNPs associated with total soybean Y, but only the locus on chromosome 14 contained a candidate gene with a well-supported biological function [64]. *Glyma.14g009900*, a homolog of *Arabidopsis thaliana* At5g19855, encodes the RbcX protein, which is involved in the assembly of Rubisco (Ribulose-1,5-bisphosphate carboxylase/oxygenase), the enzyme responsible for the photosynthetic fixation of carbon dioxide [131]. This makes the *Glyma.14g009900* a promising candidate gene whose effect on Y may be related to the regulation of photosynthetic efficiency and biomass accumulation rather than the direct control of yield components.

Loci associated with high productivity under well-watered conditions often lose their significance under stress conditions, whereas adaptive alleles that help maintain yield under drought may not be expressed in a favorable environment [37,90]. This pattern is supported by GWAS results obtained directly under drought conditions. For example, a study identified five QTNs, located on chromosomes 4, 6, 8, and 18, associated with Y under water deficit [67]. These findings indicate limited overlap between the genomic regions controlling yield under optimal water supply and under drought. Consequently, the genetic control of this trait under stress conditions only partially overlaps with that of productivity under optimal water supply. This confirms the need for dedicated GWAS studies under water deficit to identify the loci underlying plant adaptation to drought.

### 6.2. Seed Weight per Plant and 100-Seed Weight

According to published data on SoyBase [51], approximately 396 QTLs associated with seed weight/size have been identified [132]. Among yield components, SWP and HSW are of particular importance. Both traits are key indicators of soybean productivity, economic value, and seed quality, and they also play an important role in the evolutionary adaptation of crops [121,133,134].

Findings from various studies indicate that the loci controlling SWP are distributed across most chromosomes of the soybean genome. In a study involving 211 soybean accessions, 13 SNPs associated with SWP were identified, located on ten chromosomes [61]. Despite the substantial number of associations detected, no candidate genes were proposed for any of these loci. In contrast, in their analysis of HSW, the same authors identified 19 statistically significant SNPs, four of which had associated candidate genes. Notably, these genes were also associated with other yield components, such as PNP and NSP, suggesting a possible pleiotropic role in soybean productivity. In a GWAS with 309 soybean accessions genotyped on the SoySNP50K platform, 22 statistically significant SNPs associated with SW were identified. These SNPs were distributed across 12 of the 20 soybean chromosomes, with most located on chromosomes 4, 6, 14, and 19. The authors also showed that each identified locus explained less than 4% of the phenotypic variation, confirming the polygenic inheritance of SW [135]. Similar results were obtained in studies conducted under contrasting water regimes. SNPs associated with SWP were predominantly located on chromosomes 6, 14, and 20 [67], pointing to an important role for these genomic regions in the genetic control of this trait. In a study conducted under both optimal water supply and drought [68], 6 and 30 significant SNPs, respectively, were identified for SWP. Under optimal water supply, the loci were located on chromosomes 1, 3, 4, and 20, whereas under drought most SNPs were concentrated on chromosomes 4, 8, and 13. Additionally, SNPs associated with SW were detected on soybean chromosomes 14, 15, and 16 [88]. Similar studies have also identified loci associated with this trait. For example, an analysis of SWP identified six statistically significant SNPs [64]. However, a candidate gene *Glyma.18g297900* (GO:0005488), encoding a HEAT repeat-containing protein, was proposed for only one of them, *ss715632502*, on chromosome 18. The same study identified eight SNPs associated with HSW, but only *Glyma.09g171300* was proposed as a candidate gene for this trait. This gene is located near SNP ss715603826 on chromosome 9, which had previously been associated with soybean seed formation [136]. This genomic region also coincides with the previously described QTL Seed weight [137]. *Glyma.09g171300* encodes subunit 8 of the cytochrome b6/f complex, which is involved in electron transport during photosynthesis. Changes in the activity of this complex have been shown to affect plant yield [138]. In contrast to the previous study, a substantial number of associated SNPs were located on chromosome 10, suggesting that this region may harbor important loci controlling HSW [63]. In a population of 147 recombinant inbred lines (RILs) derived from a cross between the cultivars Charleston and Dongnong 594, two QTLs associated with SWP and four candidate genes located on chromosome 1 were identified [44]. However, the functional role of these genes in controlling HSW requires further investigation. In another study using a RIL population derived from a cross between lines PI595843 and WH, 38 QTLs significantly associated with HSW were identified, including four loci with a large phenotypic effect [139].

Despite the large number of QTLs identified, only a few genes controlling HSW have been functionally validated. A GWAS analysis of a panel of 1714 soybean accessions identified 35 stable loci associated with HSW. In addition, combining GWAS with linkage mapping identified the gene *GmSW17.1*, whose *GmSW17.1T* allele is associated with increased HSW. This allele results from an SNP (*Chr17:8548439 T/C)* located in the second exon of *Glyma.17g109100* [132]. Similar results were obtained in other studies, which identified a candidate gene on chromosome 17 associated with HSW [140]. Together, these findings point to the importance of this genomic region in controlling the trait. The positive correlation between HSW and other yield components, including the PNP and NSP, further suggests an interconnected genetic control of soybean productivity [31,141]. At the same time, the limited functional validation of the identified loci and candidate genes remains one of the main obstacles to their practical application in breeding.

### 6.3. Pod Number per Plant, PNP and Number of Seeds per Pod, NSP

The genetic architecture of pod-related traits in soybean has been investigated in numerous studies based on genetic linkage maps constructed from populations derived from two or more parents [142,143,144]. The PNP and SWP, as well as seed size and weight, have been shown to be closely linked to soybean yield and to play a key role in its formation [145,146]. The SoyBase database [51] currently lists more than 500 QTLs associated with pod-related traits, distributed across all 20 soybean chromosomes. For example, using composite interval mapping in a population of 207 F_2_–F_3_ progeny derived from a cross between *CS3035P-TA276-1-5-2* and *UFVS2012*, 30 QTLs associated with pod traits were identified [147]. Of these, four QTLs with pronounced additive effects were recommended for use in MAS programs.

Several studies of the genetic architecture of yield components have used genetically diverse soybean panels that included landraces and wild forms. For instance, 191, 113, and 809 genotypes were examined [115,122,148]. One QTL was identified on chromosome 6 associated with PNP and nodes [117], whereas other loci associated with PNP were detected on chromosomes 3, 5, and 6 [122,148]. Notably, some of the identified loci coincided with regions detected in the SoyNAM population, which comprised approximately 5600 lines from 40 biparental populations sharing a common parent, evaluated across six environments over three years [30]. This finding confirms the reproducibility of the results and points to an important role for these genomic regions in the genetic control of soybean yield components. Loci associated with PNP and NSP were primarily located on chromosomes 20 and 15, respectively [61]. In another study, five candidate genes, *Glyma.06g153500*, *Glyma.06g153400*, *Glyma.02g065600*, *Glyma.08g018500* and *Glyma.10g065700*, were identified near significant QTNs for PNP. Their functions are related to transport of various compounds, maintenance of genome integrity, plant growth and development. The same study identified three candidate genes, *Glyma.01g007800*, *Glyma.06g321300* and *Glyma.12g083700*, for NSP. Functional annotation showed that these genes were involved in the epigenetic regulation of gene expression and cell cycle regulation, suggesting a potential role in plant productivity [149]. For NSP, findings revealed that SNP *Chr13:27800164* [65], detected at both sowing densities, coincided with the HSW genetic region [132], and was one of 19 SNPs located within previously mapped QTL regions [148,150]. This suggests that the identified locus may be involved in the genetic control of both HSW and the NSP.

In another study conducted under contrasting water regimes, 7 and 15 significant SNPs associated with NSP were identified under optimal water supply and drought, respectively [68]. Under optimal water supply, the loci were located on chromosomes 4 and 19, whereas under drought all 15 SNPs were located exclusively on chromosome 8. Under similar experimental conditions, SNPs associated with NPN were located on chromosome 3 [69] and chromosome 5 [67].

Thus, the results of GWAS indicate a complex genetic control underlying pod- and seed-related traits in soybean. Although a large number of loci have been identified, only some have been confirmed across independent populations and different growing conditions. This shows the substantial influence of genetic background and environmental factors. At the same time, the identification of stable loci and candidate genes provides a basis for developing diagnostic molecular markers with their application in marker-assisted selection of high-yielding soybean cultivars.

## 7. Functional Interpretation of Candidate Genes Associated with Soybean Yield

GWAS is an effective approach for identifying the genetic basis of phenotypic variation and relies on the joint analysis of genotypic and phenotypic data from large, genetically diverse populations. Phenotyping can be performed across different years, under different environmental conditions, or under contrasting growing conditions, such as adequate watering versus water deficit. Genotype × environment (G × E) interaction and the environment-dependence of associated loci are important considerations when interpreting GWAS results in plants [151,152].

The primary outcome of GWAS is a set of statistically significant marker-trait associations, MTAs, which allow genomic regions associated with the trait of interest to be localized. However, a detected association does not prove the direct involvement of a specific SNP, or of a nearby gene, in controlling the trait. Because of linkage disequilibrium, LD, a single GWAS signal may correspond to several correlated genetic variants [153]. Therefore, the identified loci should be considered as potential genomic regions for candidate gene discovery rather than as experimentally confirmed causal factors. Identifying and prioritising the most probable candidate genes requires further investigation, including analysis of the genomic region surrounding the significant marker, functional annotation, expression analysis, haplotype analysis, and experimental validation [151].

In the publications that formed the core literature for this review, considerable attention is given to identifying and selecting candidate genes potentially associated with the trait of interest after GWAS. As these studies show, one selection criterion is the reproducibility of the association between a genetic variant and the trait across different conditions [61,62,65,140]. In particular, detecting the same association in several growing conditions or environments within a single study can provide additional support for the robustness of the signal. This should be distinguished from replication in independent experiments or studies, where the same locus or association is detected in different populations, experiments or datasets. Other selection criteria include detection of the association by multiple GWAS models, physical proximity of the gene to the significant marker [61] and the statistical significance of the identified SNP [63,64].

An important step after a significant genetic association is identified is to define the corresponding genomic region and analyze the candidate genes located within it. In the published studies, genomic intervals of 100 to 670 kb around the significant association were examined to search for potential candidate genes [61,62]. This approach narrows the search area and identifies genes located close to the associated marker. However, physical proximity alone does not confirm a gene’s functional role. Candidate selection therefore takes into account functional annotation, predicted biological function and information on orthologs in *Arabidopsis thaliana*, which helps assess the gene’s possible involvement in biological processes related to the trait of interest.

To further evaluate the link between a candidate gene and the trait of interest, some studies analyzed its expression [61,65,67,69,140], and a few examined its co-expression with other genes involved in the relevant biological processes [126]. Several studies also used haplotype analysis [61,65,68,124,135,140] as an additional level of genetic support for the association between candidate genes and the trait. These approaches strengthen the evidence, but they do not by themselves establish the causal role of a particular gene.

In most studies, the main purpose of identifying genetic associations is their subsequent use in breeding programs. However, only a few studies have carried this process through to the development and practical application of molecular markers for marker-assisted selection (MAS) [65]. The authors of these studies also emphasize the need for further validation of the identified candidate genes and associated markers to confirm their reproducibility and practical value in breeding programs.

Despite the widespread application of SNP-based GWAS, this approach has certain limitations. The analysis of individual SNPs may fail to capture interactions among genetic variants, including those located within the same gene. This is particularly relevant for complex quantitative traits, whose genetic control is determined by the combined effects of multiple genes, each contributing a relatively small proportion of the phenotypic variation. Moreover, the expression of such traits may be strongly influenced by environmental conditions, which is particularly characteristic of traits related to drought tolerance and yield components [151,153].

In this context, the absence of a strong statistical association for an individual SNP does not necessarily indicate low biological significance of the corresponding genetic variant. In the genetic control of complex quantitative traits, even variants with small effects may contribute to phenotypic variation, particularly through their combined effects with other genetic factors [154]. Therefore, in the present review, GWAS results are additionally considered at the level of candidate gene sets and their functional relationships.

**Figure 4:**
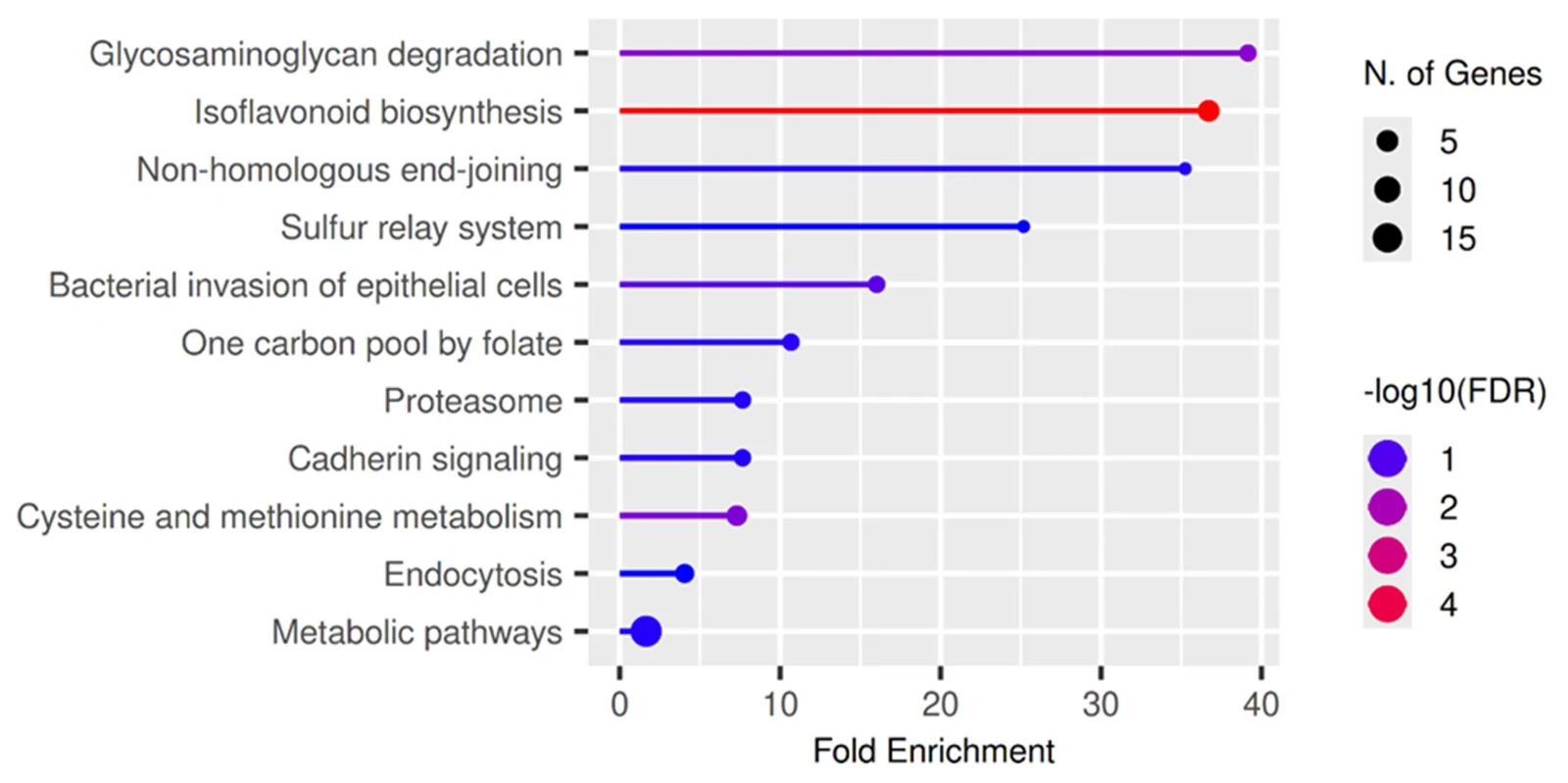
KEGG pathway enrichment analysis of soybean candidate genes.

In this study, 229 candidate genes (Supplementary Table S1) selected on the basis of the published literature were analyzed using KEGG [72] to identify shared biological pathways and relationships among the genes. Of the 229 identified candidate genes, the largest number was associated with Y, with 88 candidate genes, followed by HSW with 69 candidate genes. The smallest number of candidate genes was identified for NSP, with 21 candidate genes. Of the 229 candidate genes, 218 were identified in the database, of which 150 genes (69%) were successfully mapped. No corresponding database entries could be found for the remaining 68 candidate genes. This analysis made it possible to determine the biological pathways in which the studied genes were predominantly represented and to identify potentially significant metabolic processes associated with soybean adaptation to water deficit (Figure 4).

The strongest enrichment was found for the isoflavonoid biosynthesis pathway (gmx00943), which included five candidate genes identified from soybean yield GWAS. The enrichment level was 36.72-fold, with an FDR of 1.33 × 10^−5^ (Table 1), indicating a statistically significant and disproportionately high representation of candidate genes within this metabolic pathway. This result points to a potential link between isoflavonoid metabolism and the mechanisms underlying soybean adaptation to water deficit. This link between isoflavonoid metabolism and water stress is supported by the literature. It was shown that prolonged, progressive water deficit during seed development reduces isoflavonoid content in soybean seeds, with the degree of reduction depending on stress intensity and genotype [155]. At the expression level, the *IFS2* gene was found to be down-regulated under prolonged water deficit and to be closely associated with isoflavonoid accumulation. It suggests its potential contribution to the reduction in isoflavonoid content under drought.

Among the identified genes, *Glyma.09g048700*, which belongs to the *GmCYP81E* cytochrome P450 family, is involved in isoflavonoid biosynthesis and is annotated as isoflavone 2′-hydroxylase (EC: 1.14.14.90/1.14.14.89). This enzyme catalyzes the hydroxylation of isoflavones to form 2′-hydroxylated derivatives. In a study of γ-ray-induced soybean mutants, the expression of *Glyma.09g048700* varied depending on genotype and stress exposure; in particular, its expression decreased under PEG-induced stress in the SM1 mutant line. These findings suggest a possible role for *Glyma.09g048700* in the reprogramming of isoflavonoid metabolism under stress conditions [156,157].

*Glyma.09g048900* encodes a cytochrome P450 CYP81 family protein and is annotated in current genome databases as isoflavone 2′-hydroxylase/isoflavone 2′-monooxygenase. This gene is also of interest as a potential candidate, as it was localized in close proximity to a stable yield QTL on chromosome 9 detected across multiple environments [68,158]. However, its direct involvement in controlling yield or drought tolerance has not been experimentally confirmed. Three other candidate genes (*Glyma.09g048800*, *Glyma.09g049100*, and *Glyma.09g049200*) also belong to the CYP81 family, members of which are involved in the modification of secondary metabolites, including isoflavonoids. *Glyma.09g048800* shows high expression in root tissue, suggesting a potential role in root-related processes. However, the available evidence primarily links it to defense responses against soil-borne pathogens, so a direct link between this gene and drought tolerance should be regarded as hypothetical. *Glyma.09g049100* is annotated as *CYP81E11* (isoflavone 2′-hydroxylase), whereas *Glyma.09g049200* belongs to *CYP81E10* and is associated with isoflavonoid secondary metabolism. In addition, *Glyma.09g049200* and *Glyma.09g048900* were identified among *CYP81* genes positively correlated with *IFS2* expression within a co-expression network linked to soybean phytoalexin biosynthesis [159].

KEGG enrichment analysis also identified the glycosaminoglycan degradation pathway (gmx00531), represented by two candidate genes, *Glyma.13g163000* and *Glyma.10g091000*. This pathway includes processes of the degradation of various glycosaminoglycans, including heparan sulfate, chondroitin sulfate, dermatan sulfate, and keratan sulfate (KEGG: Glycosaminoglycan degradation). Although this KEGG pathway is primarily described for animal systems, the detection of corresponding annotations in soybean may reflect conserved enzymatic mechanisms of polysaccharide metabolism. The potential biological relevance of this finding in the context of drought tolerance may be related to cell wall remodeling processes. In soybean, water deficit has been shown to cause changes in the metabolism of cell wall components, and remodeling of cell wall structure may help maintain water balance and support plant adaptation to drought. In particular, drought-stressed *G. soja* showed enhanced metabolism of pentose, glucuronate, and galactose, associated with cell wall remodeling and an increased capacity for water uptake [159]. Thus, the enrichment of the glycosaminoglycan degradation pathway among the candidate genes may indicate a potential role for polysaccharide metabolism and remodeling processes in soybean adaptation to water deficit. However, the direct involvement of *Glyma.13g163000* and *Glyma.10g091000* in drought tolerance needs to be confirmed by further functional and expression studies.

KEGG enrichment analysis also identified the Cysteine and methionine metabolism pathway (gmx00270), which was associated with four candidate genes: *Glyma.06G178200*, *Glyma.01G007800*, *Glyma.14G111800*, and *Glyma.08G261200*, involved in the corresponding metabolic and cellular processes. The enrichment of the Cysteine and methionine metabolism pathway is of particular interest, as these metabolic processes are linked to soybean drought adaptation. Data in the literature showed that drought-tolerant soybean genotypes are characterized by a reprogramming of amino acid metabolism, including cysteine and methionine metabolism. It may help maintain redox balance and nitrogen metabolism under water deficit [160].

The remaining identified pathways showed less significant FDR values (Table 1). Enrichment of the proteasome pathway gmx03050 (*Glyma.17g252100* and *Glyma.17g253600*) may be relevant to drought adaptation. Because proteasome-mediated protein degradation contributes to the removal of damaged proteins and the maintenance of cellular homeostasis under stress. Authors have reported increased expression of *PAE1* in soybean, which encodes the α-subunit of the 20S proteasome, specifically in a drought-tolerant transgenic genotype. It suggests a possible role for proteasomal proteolysis in drought adaptation [161]. The involvement of the ubiquitin–proteasome system in soybean stress responses is also discussed in the review [162].

Overall, the functional annotation results provide additional information on the potential biological functions of the candidate genes and help prioritize individual candidates for further study. These genes can be regarded as targets for subsequent experimental validation, including confirming their association with drought tolerance and assessing the feasibility of developing SNP markers for use in marker-assisted selection.

## 8. Conclusions and Future Research Directions

Further improvements in the effectiveness of GWAS will depend on several factors, including the use of larger and more genetically diverse populations, advances in high-throughput phenotyping methods, and the application of modern statistical models. Additional gains are expected from integrating GWAS with multi-omics approaches and from the functional validation of candidate genes. Studies on regional soybean collections, including genetic resources from Central Asia, are of particular relevance. Such studies could broaden the range of available adaptive alleles and improve the efficiency of breeding cultivars with high and stable yield.

The literature analysis conducted in this review shows that GWAS is an effective tool for studying the genetic control of soybean yield and its components. A large number of genetic associations and putative candidate genes have been identified. However, many of these associations are environment-dependent and require further validation. In particular, associations identified under drought conditions should be distinguished from associations detected under well-watered conditions and should not automatically be interpreted as drought-tolerance loci. Although overlapping chromosomal regions were observed across some studies, the same marker–trait associations were not consistently reproduced across independent studies. GWAS results make it possible to identify genomic regions and loci that are statistically associated with the traits under study, but they do not directly confirm the causal role of a specific gene. Candidate genes identified based on physical proximity or functional annotation should therefore be regarded as putative candidates unless supported by independent molecular or functional evidence. Most studies of the genetic basis of soybean yield have been conducted under optimal water supply. By contrast, the genetic mechanisms underlying the maintenance of productivity under drought remain insufficiently studied. Further research should focus on identifying genetic associations that are stable across environmental conditions and on evaluating genotype × environment interactions. The chromosome 8 region associated with PNP and other highlighted genomic regions may represent targets for further investigation; however, their potential breeding value should be evaluated in relation to the population studied, environmental conditions, effect size, and independent validation rather than considered as established breeding targets. Integrating GWAS with high-throughput phenotyping, transcriptomic analysis, and genomic selection may improve the prediction of breeding value and support the development of high-yielding, drought-tolerant soybean cultivars adapted to limited water availability.

## Figures and Tables

**Table 1:** Results of the KEGG biological pathway enrichment analysis for the candidate genes. FDR: false discovery rate; FDR < 0.05 indicates statistically significant enrichment.

FDR	Genes	Pathway Genes	Fold Enrichment	Pathway
2.97 × 10^−2^	2	18	39.16	Glycosaminoglycan degradation
1.33 × 10^−5^	5	48	36.72	Isoflavonoid biosynthesis
1.57 × 10^−1^	1	10	35.25	Non-homologous end-joining
1.77 × 10^−1^	1	14	25.18	Sulfur relay system
8.74 × 10^−2^	2	44	16.02	Bacterial invasion of epithelial cells
1.52 × 10^−1^	2	66	10.68	One carbon pool by folate
1.57 × 10^−1^	2	92	7.66	Proteasome
1.57 × 10^−1^	2	92	7.66	Cadherin signaling
3.84 × 10^−2^	4	193	7.31	Cysteine and methionine metabolism
1.77 × 10^−1^	3	261	4.05	Endocytosis
1.57 × 10^−1^	18	3842	1.65	Metabolic pathways

## Data Availability

The original contributions presented in this study are included in the research paper and in Supplementary Materials. Further inquiries can be directed to the corresponding authors.

## Supplementary Materials

The following supporting information can be downloaded at https://www.mdpi.com/article/10.3390/plants15203243/s1. Table S1: Candidate genes associated with soybean yield and yield-related traits and their functional annotations. Table S2: Details of molecular marker distribution and genetic loci based on the current analysis across 20 chromosomes in soybean (*Glycine max* L.).
